# Management of Equine Post-Extraction Cheek Tooth Alveoli: Application of Alveolar Plugs

**DOI:** 10.3390/ani16111678

**Published:** 2026-05-30

**Authors:** Joanna Śmich, Kamil Górski, Małgorzata Maśko, Marta Borowska, Bernard Turek, Małgorzata Domino

**Affiliations:** 1 Department of Large Animal Diseases and Clinic, Institute of Veterinary Medicine, Warsaw University of Life Sciences, 02-787 Warsaw, Poland; joanna_smich@sggw.edu.pl (J.Ś.); kamil_gorski@sggw.edu.pl (K.G.); bernard_turek@sggw.edu.pl (B.T.); 2 Department of Animal Breeding, Institute of Animal Science, Warsaw University of Life Sciences, 02-787 Warsaw, Poland; 3 Institute of Biomedical Engineering, Faculty of Mechanical Engineering, Białystok University of Technology, 15-351 Białystok, Poland; m.borowska@pb.edu.pl

**Keywords:** premolar teeth, molar teeth, tooth extraction, exodontia, filling material, dental disease, equine dentistry, horse

## Abstract

Preservation of dentition remains the primary goal of equine dentistry; however, cheek tooth extraction is required in cases of severe dental pathology. Following tooth extraction, management of the post-extraction alveolus remains a significant clinical challenge due to its large size and susceptibility to contamination with feed material and bacteria. To mitigate these risks, alveolar plugs are commonly used to protect the alveolus, stabilize the blood clot, and support granulation tissue formation. This review summarizes the current case reports and research articles regarding the use of alveolar plugs following equine cheek tooth extraction, with a focus on packing materials, post-extraction management strategies, complications, and clinical outcomes. Reported packing materials include polymethyl methacrylate (PMMA), plaster of Paris (PoP), dental wax, gauze swabs, and polyvinyl siloxane (PVS). Each material has unique advantages and disadvantages, which are summarized in this review. The choice of plug material and its management protocol should be individualized for each case, as it depends on alveolar depth, tooth location, extraction method, and the presence of complications. Although standardized protocols for equine alveolar plug management have not yet been established, it may be suggested that any of the currently described packing materials can be used following routine tooth extractions. For marginally positioned teeth, gauze swab plugs may be more favorable, although they may not be the optimal choice in older horses. Regardless of whether the extraction is routine or complicated, alveolar inspection at 7–14-day intervals may be recommended. However, specific recommendations regarding alveolar inspection intervals and detailed management strategies require further research.

## Simple Summary

Equine alveolar plugs are materials placed into the alveolus following cheek tooth extraction to protect the site and promote healing. In horses, commonly used alveolar plug materials include polymethyl methacrylate (PMMA), plaster of Paris (PoP), dental wax, gauze swabs, and polyvinyl siloxane (PVS). Equine alveoli are deep and relatively large, and when left open, they are susceptible to contamination by food material and bacteria. Therefore, alveolar plugs help protect the alveolus against contamination, stabilize the blood clot, reduce infection risk, and support granulation tissue formation necessary for alveolar healing. However, as optimal recommendations for the use of alveolar plugs in horses have not yet been clearly established, this narrative review aims to summarize the existing evidence regarding the application of alveolar plugs following equine cheek tooth extraction. Considering clinical significance of using alveolar plugs, this review summarizes the advantages and disadvantages of specific packing materials, presents examples of their clinical application, discusses clinical outcomes of post-extraction alveolar healing with plugs, and proposes general recommendations for the management of post-extraction alveoli following both routine and complicated extractions. Overall, this review highlights an important role of alveolar plugs in equine alveolar healing, while also identifying gaps in the current literature, including the lack of standardized protocols and the need for further research to determine optimal plug management strategies.

## 1. Introduction

The average lifespan of equine companion animals has significantly increased, largely attributed to improved veterinary care, advancements in husbandry practices, and owner education about preventative health and potential diseases [1]. Owner interest in dental diseases and treatments has also gained attention over the years [2,3]. Maintaining dentition is the primary objective of dentistry; however, tooth extraction referred to as exodontia, may sometimes represent the most appropriate treatment option for certain clinical cases. Among oral surgical procedures, tooth extraction, is one of the procedures most commonly performed in humans [4] as well as domesticated animals, such as dogs, cats, horses, rodents, or rabbits [5,6,7].

Tooth extraction is the preferred treatment for dental diseases in horses [8,9,10], and it is typically used as a last resort for fractures (idiopathic or related to infections), dislocation, maleruption/impaction, excessive mobility, caries, or the presence of severe diastemata causing periodontal pain [8,10,11]. According to a recent study, apical tooth infection was the primary indication for cheek tooth extraction, accounting for 62% of 428 extractions [9]. This finding is supported by the numerous clinical evidence, in which apical infection [8,10,11,12,13,14,15] and sinusitis [14,15,16,17,18] were commonly reported reasons for extraction procedures. Other reported indications included dental displacement [8,10], fractures of the tooth [8,10,18] or bone (particularly the mandible) [16], bone sequestration [16], oral ulceration [8], periodontal infection [8,11,15], diastemata [10], as well as the presence of fistulae [12,16], supernumerary cheek teeth [10,19], or hypoplastic teeth [20]. Some horses were also referred for extraction because of difficulties encountered by the referring practitioner in the field [9,21], failure of oral extraction [7], or the presence of retained tooth root fragments within the alveolus [16,22] or sinus [18]. Only one prospective study involved experimentally performed extractions to evaluate the effect of an alveolar bone substitute on post-extraction drift of maxillary cheek teeth [23].

In horses, tooth extraction is a challenge, as is ensuring proper healing of the dental alveolus. The literature suggests a lack of consensus regarding the types of alveolar plugs used in horses and their application in specific clinical cases. There is also a lack of reliable data on the frequency of their replacement [9,21,24]. Each veterinarian performing tooth extractions in horses has their own well-thought-out protocol for the selection and frequency of alveolar plug replacement. Therefore, this narrative review aims to summarize existing evidence on the types and use of alveolar plugs following equine cheek tooth extraction, focusing on plug management, clinical outcomes including the normal healing process and complications, as well as current recommendations for the management of the equine alveolus post extraction.

## 2. Literature Search Strategy

This narrative review was based on a literature search conducted in 2025 using the PUBMED, Scopus, and Web of Knowledge electronic databases. The searching strategy included in PUBMED (#1: alveolar bone*[tw] OR alveolus*[tw]; #2: extraction*[tw] OR healing[tw]; #3: horse*[tw] OR equine*[tw]; #4: #1 AND #2 AND #3), in Scopus (alveolar bone* OR alveolus*) AND (extraction* OR healing) AND (horse* OR equine*), and in Web of Knowledge (#1: TS = (alveolar bone* OR alveolus*); #2: TS = (extraction* OR healing); #3: TS = (horse* OR equine*); #4: #1 AND #2), resulting in 33, 88, and 58 retrieved records, respectively.

The inclusion criteria for this narrative review encompassed case reports and research articles on the alveolar healing process, involving the use of plug materials, following extraction of equine cheek teeth, published from 1975 onwards. The exclusion criteria included lack of full-text availability in English and absence of an available abstract. Case reports and research articles were summarized in the main table, whereas conference communications, reviews, and book chapters were excluded from the summary table; however, they were used to support the discussion when the required information was lacking in the case reports and research articles.

For the selection process, a total of 179 retrieved records were compiled into an Excel file. Duplicates were manually removed and after this removal, 103 records remained. Subsequently, an unblinded screening was conducted, initially based on the title and then on the abstract. In the first step, titles related to species other than horses or to aims unrelated to alveolar healing were excluded. If the species or aim could not be determined from the title, the records proceeded to the second stage of selection. The title screening step resulted in 64 remaining records. In the second step, abstracts unrelated to horses or not relevant to alveolar healing were excluded. Following abstract screening, 34 records remained and underwent full-text retrieval.

For the full-text evaluation, a data extraction sheet was used. The extracted information included: the year of publication; study type (case report, research article, review, book chapter, conference communication, or other); the level of Evidence-Based Medicine Rating (EBMR) within the hierarchy of evidence (if applicable) [25,26]; the aim of the study; the extraction type (routine or complicated); the number of horses included; the location of the extracted tooth; the number of teeth extracted; the packing material used; the eluted medication administered; the extraction method; the alveolar management; the recheck intervals; healing time; the criteria used to assess alveolar healing; and complications following tooth extraction. Routine extraction was defined as a simple oral extraction procedure performed in a standing horse or under general anesthesia. Complicated extraction was defined as a procedure requiring more traumatic techniques for tooth removal, such as repulsion, tooth sectioning, or alveolar curettage, regardless of whether it was performed in a standing horse or under general anesthesia. After analysis of the extracted data, 17 records that did not meet the inclusion criteria were excluded from the general summary, including 4 case reports unrelated to cheek tooth extraction and alveolar healing using plugs, 6 research articles unrelated to cheek tooth extraction and alveolar healing using plugs, and 7 narrative reviews. No automation tools were used in the process.

## 3. Alveolar Plugs Used for the Management of Equine Alveoli Post Extraction

Among the 17 articles included in this narrative review that described the alveolar healing process involving the use of plug materials in equine cheek teeth, 7 articles were case reports describing either single cheek tooth extraction [12,14,17,18,20,22] or double cheek teeth extraction [15] in a single horse, whereas 10 records were research articles [7,8,9,10,11,13,16,21,23,27]. The research articles included between 5 [16,23] and 400 [9] horses and predominantly consisted of retrospective clinical case series [7,8,10,11,13,16,21,27], with one retrospective cohort study [9] and one prospective cohort study [23]. Therefore, the reviewed studies were primarily clinical in nature and supported by case reports with non-applicable EBMRs [12,14,17,18,20,22], as well as research articles occupying a low position in the hierarchy of evidence [7,8,10,11,13,16,21,27], predominantly classified as level 4 EBMR [25,26].

Among the case reports, two articles described routine extraction of maxillary cheek teeth by oral extraction [14,15], while one article described extraction of a retained mandibular cheek tooth root by alveolar curettage [22]. Moreover, four articles reported extraction of maxillary [17,18,20] and mandibular [12] cheek teeth using repulsion techniques. In these case reports, repulsion methods included sinusotomy [18], osteotomy [12], and trephination [17,20].

Following each tooth extraction, the alveolus was filled with plugs composed of clinically used packing materials, such as polymethyl methacrylate (PMMA) [15], plaster of Paris (PoP) [20], gauze swabs [14,15,17,22], and polyvinyl siloxane (PVS) [12,15,17,18,20]. The case reports described are summarized in Table 1.

Among the research articles, eight described retrospective analyses of clinical cases involving extraction of maxillary [7,8,10,11,13,16,27] and mandibular [7,8,10,13,16,21,27] cheek teeth. One article was a retrospective cohort study evaluating the extraction of both maxillary and mandibular cheek teeth [9], while another was a prospective cohort study focusing exclusively on extraction of the last maxillary premolar teeth (108 and 208 according to the Triadan system [28]) [23]. Three case series described both routine and complicated extractions [8,11,21]. In the complicated cases, teeth were extracted using tooth sectioning [21] and repulsion techniques [8,11,21], including sinusotomy [8,11], trephination [8,11], buccotomy [8], and repulsion through a preexisting fistula [21]. In some cases, the specific repulsion technique was not recorded [10]. Additionally, five case series described only complicated cases [7,13,16,23,27], in which cheek teeth were extracted using tooth sectioning [27] and repulsion techniques [7,13,16,23]. These repulsion methods included sinusotomy [7,13,23], trephination [7,13,16], osteotomy [16], buccotomy [16], and repulsion through a preexisting fistula [13]. In some cases, the specific repulsion technique was also not recorded [13,16]. The retrospective cohort study included both routine and complicated extractions, with the latter involving repulsion by trephination or the use of transbuccal screws [9]. In contrast, all horses included in the prospective cohort study underwent the same complicated extraction procedure involving repulsion by sinusotomy [23].

Following tooth extraction, most alveoli were filled with plugs composed of PMMA [7,8,9,10,16,27], PoP [8,11,16], dental wax [10,13], gauze swabs [8,9,10,13,16,21], or PVS [8,13,16,21,23,27]. In only one article [8], the alveolus was reported to be left unfilled in 22 of the 115 included horse cases. The research articles described are summarized in Table 2. Given the high heterogeneity of the summarized studies, the application of alveolar plugs for the management of post-extraction cheek tooth alveoli in horses is presented descriptively below.

## 4. Plug Management

Depending on the species and clinical situation, the post-extraction incision may be closed or left open after cheek tooth extraction. It is widely acknowledged that closing the extraction wound in humans and small animals promotes wound healing, safeguards the dental alveolus, lowers the possibility of food particle contamination, which lowers the risk of infection or alveolar osteitis, and ultimately reduces pain [23,29,30]. Most frequently, interrupted resorbable sutures are used to close it [6]. Although, if the alveolus is contaminated by active infection at the time of the extraction procedure, the extraction wound is left open, allowing drainage of the infection site [31]. Contrastingly, in horses, the extraction wound is typically not sutured and is left open, allowing healing by secondary intention, including granulation tissue formation, with the exception of some reported cases following incisor extraction [32]. Furthermore, to prevent contamination of the deep and relatively large alveolus and to prevent impaction of long-stem forage in the alveolus before alveolar closure with granulation tissue, a dental plug or packing material may be inserted into the alveolus [10,12]. Among the summarized 7 case reports and 10 research articles, only one study reported alveoli being left unfilled in 22 of the 115 included horses [8]. However, in the same study [8], as well as in the remaining 16 studies [7,9,10,11,12,13,14,15,16,17,18,20,21,22,23,27], post-extraction alveoli were filled with packing materials. As most of these studies consisted of descriptions of clinical cases, it may be reasonably concluded that the use of alveolar plugs is considered clinically necessary following cheek tooth extraction in horses.

The alveolar site exhibits favorable post-extraction healing if the alveolus does not exhibit significant alveolar bone infection prior to tooth extraction, if the alveolus did not sustain excessive damage during the extraction procedure, if there are no leftover dental fragments or sequestrae, and in the presence of a large post-extraction blood clot [33]. Protection of the post-extraction blood clot from mastication forces and food impaction through the use of different types of packing material may be critical for optimal and complete healing of the alveolus. As suggested by previous studies, the alveolar plug should be placed in the more occlusal portion of the alveolus, specifically the coronal one-third, however should not extend beyond the level of the gingiva, so as not to interfere with mastication [10,16,17,20]. However, there is no strong scientific evidence supporting this clinical recommendation, as all recent studies discussing the optimal depth of plug placement consist of either case reports [17,20] or case series [10,16]. Therefore, further research is required to determine the optimal depth of plug placement at the different stages of the alveolar healing process. To set the appropriate position of the plug, Kamps and Barakzai [7] used the repulsion pin to push the plug up or down in the alveolus, only to fill 2–3 cm of the entire alveolus. This management characteristic is significant, as the space left in the more apical part of the alveolus allows for the blood clot to be replaced by granulation tissue, which gradually fills the rest of the alveolus, and also in this case, the iatrogenically created fistula, generating a seal [7].

Following cheek tooth extraction in horses, it has been suggested to protect the alveolus and alveolar blood clot from mastication forces and excessive contamination by feed and bacteria. Therefore, as standard post-extraction management, it is recommended to flush the alveolus using mild-pressure water [15,16,21], normal saline [12,20], or Hartmann’s solution [7] to remove any remaining fragments, debris, or pus and then to insert an alveolar plug into the alveolus. Similarly, gentle flushing of the healing alveoli was described during post-extraction rechecks [9,10,12,14,16,20], especially when a fistula was present [10,20]. However, a standardized management protocol has not yet been defined for the post-extraction alveolus, specifically plug effectiveness and replacement. Overall, a comprehensive review of equine alveolar plugs is required given the variability in reported techniques and clinical outcomes recorded thus far.

### 4.1. Packing Material Characteristics

A packing material is considered ideal for use as an alveolar plug if it does not impede healing and effectively seals the alveolus [9,10]. Additionally, the plug should occupy no more than half of the alveolar space, as its oversizing may inhibit granulation tissue formation or the plug may displace into the paranasal sinus [17,24,34]. A plug fails to fulfill its function if it does not adequately fill the alveolus or if its shape does not conform to that of the alveolar cavity [16], given that the function of a properly formed and situated alveolar plug is to protect the alveolus, stabilize the blood clot, reduce infection risk, and support proper healing of the alveolus [10,16,21].

The types of packing materials used as alveolar plugs following extraction of equine cheek teeth include: PMMA [7,8,10], PoP [8,11,20], dental wax [9,10,13], gauze swabs [8,9,10,14,21,22], and PVS [9,13,16,18,20,21,23,33,34,35,36,37]. The packing materials and their characteristics are summarized in Table 3. One may observe that certain packing materials—such as PMMA, PoP, or gauze swabs—may be impregnated or mixed with medications that will be released into the alveolar environment—especially antimicrobials or substances that accelerate granulation [9,10,14,15,16,21,22,24]. The second clinically important property of packing materials is biodegradability, as this allows the packing material to be gradually replaced by granulation tissue, reduces the need for manual removal, and minimizes the risk of foreign body reactions [11,38,39]. Among the main advantages and disadvantages of individual packing materials, the ability of a plug to elute drugs and biodegradability are highlighted in separate columns due to their importance for the healing process. Remaining advantages and disadvantages in practical clinical use are summarized in the next two columns.

**Figure 1:**
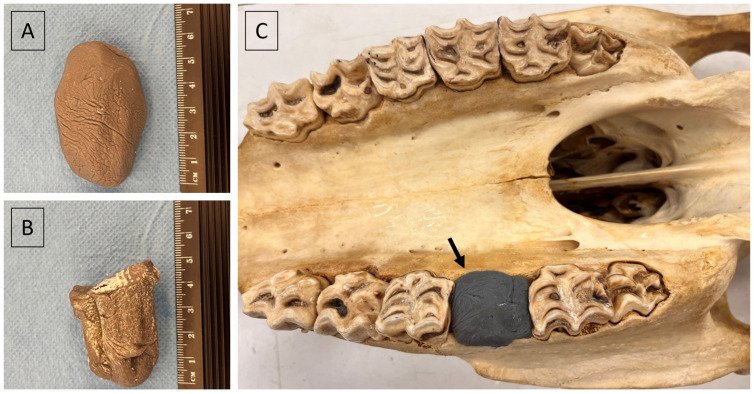
Illustration of polymethyl methacrylate (PMMA) used as alveolar plugs in equine dentistry. (**A**) PMMA plug before positioning in the alveolus and (**B**) after its removal. (**C**) Visualization of alveolar filling using a PMMA plug. The plug is marked by an arrow.

PMMA is a type of synthetic polymer that has been utilized for stabilization of fractured cheek teeth and as an alveolar plug following tooth extraction in horses. It is capable of eluting medications if added to the PMMA [11]; however, this material is not biodegradable [11]. Characteristics that make it useful as a material in equine dentistry is that it has a low density, it is affordable, and due to its pliability and quick-setting qualities, it can be structurally and mechanically adapted to the desired location in the oral cavity and provide a watertight seal of the alveolus [40]. It is a type of thermoplastic, whose curing process is exothermic [37], which may irritate and damage surrounding soft tissues and loosen prematurely. A sample PMMA plug is illustrated in Figure 1.

**Figure 2:**
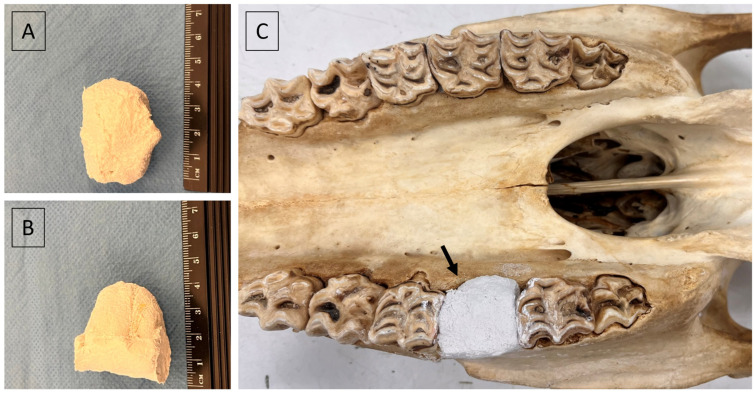
Illustration of plaster of Paris (PoP) used as alveolar plugs in equine dentistry. (**A**) PoP plug before positioning in the alveolus and (**B**) after its removal. (**C**) Visualization of alveolar filling using a PoP plug. The plug is marked by an arrow.

PoP is a simple, traditional gypsum plaster containing a white powder (calcium sulfate hemihydrate [CaSO_4_]) which is mixed with water and allowed to dry. It is cheap, stable, and quick-setting; however, it does take a longer period to harden or set compared to PMMA. This material has a capacity to elute medications [11,41]. Importantly, it is cold curing and consequently well tolerated by tissues [42]. PoP is more cost-effective than dental wax and PMMA. This material is biodegradable [11,41]. Sample PoP plug is illustrated in Figure 2.

**Figure 3:**
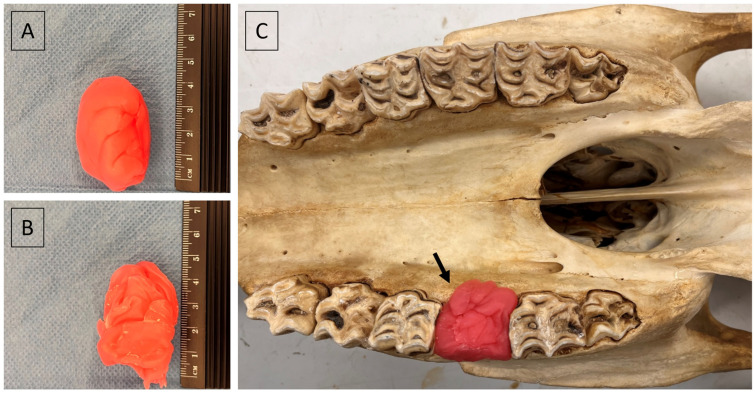
Illustration of dental wax used as alveolar plugs in equine dentistry. (**A**) Dental wax plug before positioning in the alveolus and (**B**) after its removal. (**C**) Visualization of alveolar filling using a dental wax plug. The plug is marked by an arrow.

Dental wax (paraffin, bees wax, Carnauba wax, or colorant) has also been used as a packing material for the equine alveolus, although less often compared to the other materials. It does not have the ability to elute medications [11]. It is cold curing, which is an advantageous characteristic over PMMA, which undergoes an exothermic reaction in the curing process [11]. However, this material is not biodegradable and has no capacity to elute medications [11]. A sample of a dental wax plug is illustrated in Figure 3.

**Figure 4:**
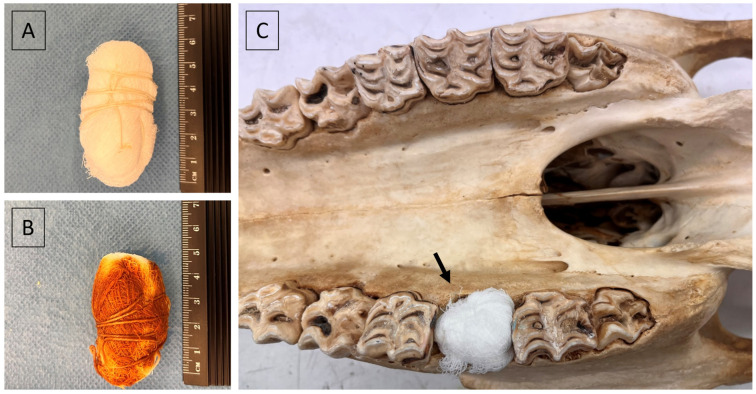
Illustration of gauze swabs used as alveolar plugs in equine dentistry. (**A**) Gauze swabs plug before positioning in the alveolus and (**B**) after its removal. (**C**) Visualization of alveolar filling using a gauze swab plug. The plug is marked by an arrow.

Gauze swabs are a simple, low-cost, and widely available packing material. It provides a means of alveolar drainage, especially in instances where the alveolus was infected or following mandibular cheek tooth extraction, where drainage is challenging due to the anatomical location of the mandibular alveolus [9]. It also facilitates the release of medications. This material does not exhibit a curing time or curing by-products [43]. It does not provide a total seal of the alveolus as PMMA or PVS packing materials and may dislodge in more shallow alveoli. Regular assessment of the alveolus is possible due to repeated replacement required with this material [9,14,21]. Gauze swabs are not biodegradable. Sample gauze swabs plug is illustrated in Figure 4.

**Figure 5:**
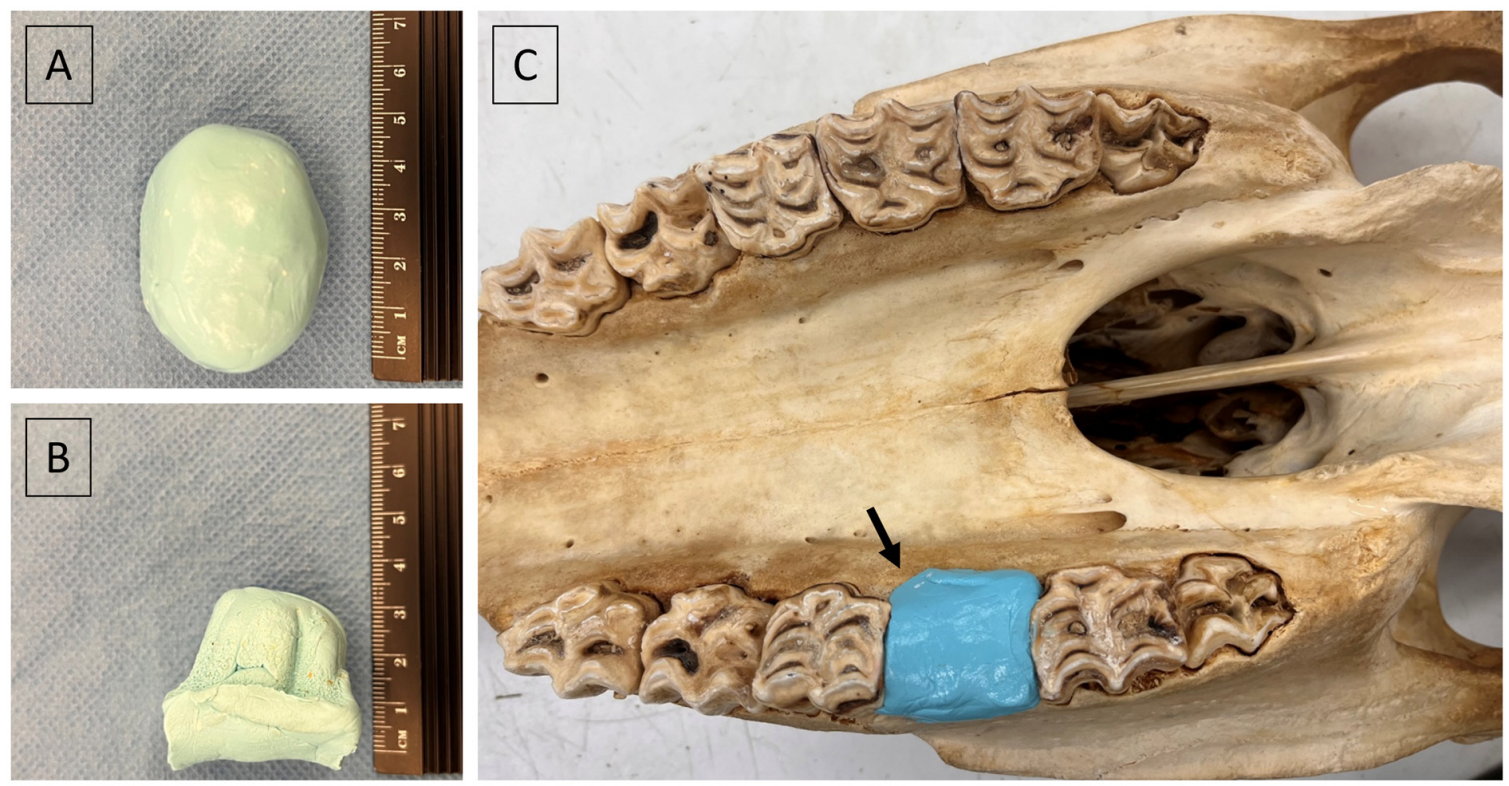
Illustration of polyvinyl siloxane (PVS) used as alveolar plugs in equine dentistry. (**A**) PVS plug before positioning in the alveolus and (**B**) after its removal. (**C**) Visualization of alveolar filling using a PVS plug. The plug is marked by an arrow.

PVS is an elastic silicone elastomer made through an addition reaction by mixing a two-part paste (a base and an accelerator) in equal quantities with the creation of no by-products [42]. It is tasteless, odorless, and flexible, making it easy to manipulate in the horse’s mouth [23,27,42]. PVS exhibits a high degree of long-term dimensional stability, showing the smallest dimensional changes in all elastometric impression materials [42]. It has a non-porous nature, serving rather as an alveolar seal than a delivery medium of medications [44]. Also, this material may be preferred due to its plastic qualities after setting [20,34]. It is not biodegradable [45]. A sample PVS plug is illustrated in Figure 5.

### 4.2. Impregnating Medications

An objective consensus for the most optimal substance to impregnate or mix with a packing material has also not yet been determined. The available literature reports the use of, e.g., antimicrobial medications, including antibiotics, synthetic chemotherapeutics, and antiseptics. This includes intra-alveolar application of metronidazole [9,10,11,21], cefazolin [11], ampicillin [9,11], cloxacillin [9], iodoform [14,20], povidone iodine [10,15], zinc oxide–iodophor–petrolatum [8], saline [22], scarlet oil [16], and medical grade honey [21]; which were locally released from the plugs. Alveolar plugs, particularly those made from PVS, were also mixed with bone substitute to accelerate the healing of the alveolus [23,35]. However, the evidence supporting the effectiveness of these impregnating medications remains limited, as their use has primarily been reported in case reports, which are not applied in the hierarchy of evidence [14,15,20,22] or case series occupying a low position in the hierarchy of evidence [8,10,11,16,21,23,35], while only one study was a retrospective cohort study [9]. Therefore, the evidence described below should be interpreted with caution.

No difference in post-extraction complications has been observed when impregnating plugs with medical grade honey compared to metronidazole and broad-spectrum antibiotics such as cefazolin, sodium ampicillin and sulbactum, and trimethoprim–sulfa or a combination of metronidazole and a cloxacillin benzathine and ampicillin intramammary suspension [9,11,21]. Therefore, in the current state of antibiotic resistance, if antibiotics are not advised, this therapy should be limited. Kennedy et al. [9] justified the use of local antibiotics incorporated into the alveolar packing material when there was pre-existing alveolar and supporting bone infection and in some cases, direct exposure of the alveolar bone during tooth extraction. Anecdotal evidence has also been recorded on the damaging effect of some antiseptic-impregnated alveolar packing materials on post-extraction alveoli and consequently, the development of alveolar sequestration [9]. In contrast, saturating alveolar plugs in concentrated antiseptic solutions, such as povidone iodine, may risk delayed healing through chemical irritation of the delicate granulation tissue [24].

### 4.3. Selecting a Packing Material for the Tooth Extraction Site

It may be suggested that alveolar plugs may be beneficial for appropriate healing of the alveolus following tooth extraction from both the maxilla and mandible. Kennedy et al. [9] and Gergeleit and Bienert-Zeit [21] suggested that maxillary alveoli have a better draining capacity due to gravity, and post-extraction complications, such as alveolar sequestration will resolve on their own or with minimal intervention. Oppositely, empty mandibular alveoli are more prone to ongoing infection, as their position provides a space for bacteria and sequesterae to remain in the alveolus [9]. Characteristically, the mandibular alveolar bone in horses may be similar to humans, providing a more demanding environment for alveolar healing compared to maxillary alveolar bone, especially after the extensive and powerful forces exerted on the alveolus during tooth extraction [9]. Thus, protecting the alveolar bone, particularly in the mandible, by properly functioning alveolar plugs, may be beneficial for efficient and proper healing of the alveolus.

Moreover, deciding on the type of alveolar plug to apply into the alveolus after each cheek tooth extraction can also depend on the Triadan number of the extracted cheek tooth [17,28]. For instance, a plug composed of any of the materials—PMMA, PoP, dental wax, gauze swabs, or PVS—is inserted into the coronal one-third of the alveolus, leaving room in the apical portion for the retention of the blood clot and granulation tissue formation. Furthermore, the adjacent teeth are dried and provide structural support and stability for the aforementioned plug materials that are placed into the empty alveolus, creating an occlusal seal. However, the function of these plugs is hindered when two adjacent teeth are removed or if the tooth that is extracted is at the very beginning or end of a dental arcade, specifically Triadan 06 or 11, respectively. In these cases, it is possible to only fix the plug to one adjacent tooth. In these circumstances, attempting to secure and stabilize the plug by inserting it deeper into the alveolus, closer to the apical aspect, will prevent the formation of granulation tissue. Consequently, in the aforementioned conditions, other gauze swab plugs may be more suitable for teeth 06 or 11, as this type of plug does not require support from an adjacent tooth. Otherwise, following routine extractions of teeth 07–10, any of the plug materials may be used. However, the alternative may be to use a three-loop wire to stabilize the marginal check tooth, as was incorporated into the PVS plug to stabilize its position [23].

### 4.4. Selecting a Packing Material Based on the Age of the Horse

Another factor to consider when selecting the type of packing material following tooth extraction is the horse’s age. Following tooth extraction in a 4-year-old horse, Stemmet et al. [17] elected to insert gauze swabs to pack the alveolus. Gergeleit and Bienert-Zeit [21] also used gauze swabs to fill the alveoli after extraction of 880 cheek teeth in horses aged between 3 and 29 years. Similarly, Kennedy et al. [9] used gauze swabs in horses ranging from 2 to 28 years; while Dixon et al. [10] primarily used gauze swabs in horses aged between 2 and 18 years; however, dental wax or PMMA were also applied.

PMMA was selected as the packing material of choice in 20 horses that underwent cheek tooth repulsion using small diameter repulsion pins. These horses ranged in age from 5 to 16 years [7]. PMMA and PVS were also used following tooth extraction by tooth sectioning in horses aged between 2 and 30 years [27]. Moreover, in a study reviewing 61 cases involving horses aged from 1 to 27 years, gauze swabs, dental wax, or PVS were inserted into the post-extraction alveoli [13]. In the study by Caramello et al. [8], involving horses aged between 1 and 27 years, alveoli were left unfilled in 22 horses, whereas gauze swabs, PMMA, or PoP were used in the remaining 115 horses. PoP was also selected as the alveolar packing material in horses ranging from 3 to 22 years of age [25]. Similarly, Turek et al. [20] used PoP to pack the alveolus in a two-year-old horse, which was later replaced with PVS due to the development of complications.

In five horses aged between 5 and 7 years, PVS was selected as the packing material of choice in a study comparing alveoli filled with a biocompatible non-resorbable bone substitute to untreated alveoli filled with a blood clot [23]. In a study by Limone and Baratt [12], an eight-year-old miniature horse also received a PVS plug. At each recheck examination, the PVS plug was removed, and the apical portion was trimmed with a scalpel before re-insertion into the alveolus. This technique allowed greater granulation tissue formation in the apical region of the alveolus [12]. PVS was also selected as the preferred packing material following cheek tooth extractions in horses aged between 4 and 21 years; factors such as the presence of an oro–sinus fistula, retained root fragments, or complicated fractures were proposed as the reasons for its selection rather than the horses’ age [16]. Similarly, in a seven-year-old horse, a PVS plug was chosen to fill the post-extraction alveolus because of the presence of an oro–sinus fistula rather than the horse’s age [18].

It may be observed that these articles did not provide further details regarding how packing materials were selected for individual cases with respect to the horse’s age. Therefore, there is currently no clear evidence in the literature supporting the selection of a specific packing material based on age. However, as horses age, the occlusal surfaces of the cheek teeth undergo progressive wear, resulting in reduced alveolar depth [46]. A shallow alveolus may decrease the stability and retention of certain plugs. Consequently, in older horses—aged ≥15 years old [47]—gauze swab plugs may be less stable and more prone to dislodgement from the alveolus. On the other hand, the difficulty in tooth extraction is likely to be different in younger and older horses, this time to the detriment of young horses [21]. Therefore, the incidence of post-extraction complications related to bone destruction, such as sequestration [7,9,10,15,21], may appear more often in younger horses, regardless of the plug materials used.

Among reported post-extraction complications, plug displacement into the oral cavity [11,16,20,23] resulted in the loss of plug function and increased the likelihood of alveolar contamination with food material. Moreover, in some cases, gauze swabs failed to prevent alveolar contamination with food material [21], excluding the consideration of gauze swabs as an ideal plug. In this specific case, the gauze swab plug was replaced with a PVS plug, although the age of the horse was not reported [21]. However, despite these risks, successful use of gauze swabs was reported in a 16-year-old pony following the extraction of retained tooth roots by alveolar curettage [22] and in a 12-year-old full size horse following routine oral extraction [14]. Consequently, alternative packing materials, such as PMMA, PoP, dental wax, or PVS, may offer advantages in selected cases involving older horses; however, the use of gauze swabs in this age group is also not excluded.

### 4.5. Retention Time of the Alveolar Plug

The optimal duration for alveolar plug retention has not yet been clearly established, as the recheck intervals and reported healing times varied among the reviewed studies. Furthermore, there is no clear consensus regarding the criteria for complete alveolar healing. One study defined the healing end-point as complete sealing of the alveolus with granulation tissue [15], whereas others considered healing complete when the granulation tissue became lined with normal gingival tissue [16,23]. However, in most articles, “normal healing” was reported without further specification [12,14,16,21,22], while in other studies alveolar healing was not reported at all [7,8,9,10,11,13,16,17,18,27]. In contrast, healing of associated fistulae was more consistently described, with fistulae considered healed when complete closure was achieved [14,18,20]. Another aspect requiring consideration is whether alveolar plugs should be manually removed [9,12,14,15,18,20,21,23] or left within the alveolus to be spontaneously displaced by the formation of granulation tissue [13,22,23].

Considering equine alveoli following routine tooth extractions, healing was reported after 6 weeks [12] and 18 months [14]. McQuillan et al. [15] performed weekly inspections of the alveolus, whereas Kau et al. [14] first examined the alveolus 4 days after extraction. Subsequently, the alveolus was rechecked every 6 days until the horse was discharged 16 days after extraction [14].

Considering equine alveoli following complicated tooth extractions, healing durations varied widely, ranging from 2 weeks [27] to 48 months [7]. Turek et al. [20] reported alveolar examinations at 3, 4, 9, and 16 days post-extraction, followed by weekly rechecks. Limone et al. [12] described examinations at 7, 21, and 42 days. Earley et al. [16] reported variable recheck intervals across different cases: in some, inspections were performed every 7–10 days; in others, at 1, 2, and 11 months; and in another case, only after 6 months. Leps et al. [27] reported the first inspection and subsequent rechecks at 2-week intervals. Kamps and Barakzai [7] described the first inspection after 2 weeks, followed by rechecks every 4–6 weeks. Horbal et al. [22] and Stemmet et al. [17] reported the first inspection after 3 weeks; however, Stemmet et al. [17] performed additional rechecks after 2 weeks and 2 months thereafter. Vlaminck et al. [23] described monthly dental examinations. Nottrott et al. [18] reported the first inspection after 10 weeks, with subsequent rechecks at 2- or 3-month intervals.

However, in studies describing both routine and complicated cases, healing duration varied widely, ranging from 2 weeks [8] to 5 [21] or several [9] months, depending on complications. Trostle et al. [11] reported alveolar examination only after 1 day, with no further rechecks. Gergeleit and Bienert-Zeit [21] reported the first inspection after 2 days, followed by weekly rechecks. Kennedy et al. [9] described alveolar inspection at 10–14 days post-extraction. Dixon et al. [10] reported alveolar inspection at 2–4 weeks for part of the cases.

This summary demonstrates that there is no standardized schedule for alveolar examination, no clearly defined recheck intervals, and no specified end-point for follow-up examinations. Furthermore, the available literature does not demonstrate a clear relationship between the type of packing material used and the recheck or healing time, nor between the method of tooth extraction and the recheck or healing time. Despite these gaps in the literature, regular examination of the healing alveolus is undoubtedly important because of the potential development of significant complications. In cases of certain complications, such as plug displacement into the sinus [13,17] or retention of intra-alveolar tooth root fragments [7,22], additional surgical procedures may be required to resolve the problem. However, when detected early, such complications may be easier to manage and less likely to result in serious long-term consequences.

## 5. Clinical Outcomes of Post-Extraction Alveolar Healing with Plugs

### 5.1. Normal Healing Process

Due to the unique anatomy of the equine dental alveolus and the relative position of the gingiva, an alveolar plug may be beneficial to protect the alveolus and support proper healing. Alveoli protected by plugs provide favorable conditions for healing by secondary intention. Alveolar plugs are also advantageous in situations where the alveolus does not want to heal completely.

Alveolar healing constitutes the histological changes that occur in the alveolus from the time of dental extraction until the complete healing and restoration of the alveolus [48]. According to human literature, this process is made up of four stages: hemostasis and coagulation, inflammatory, proliferative, and remodeling stages [49,50]. In humans, these stages progress relatively quickly with the formation of new lamellar bone and marrow [48,49]. Thereafter, the rate of new bone remodeling slows down and can last for years following dental extraction [49]. First-stage hemostasis and coagulation occurs within the first 24 h after extraction [49]. The alveolus fills with blood which develops into a blood clot, resulting in hemostasis. The blood clot is rich in red blood cells, white blood cells, and platelets within fibrin layering [49]. The blood clot is replaced by granulation tissue within the first 7 days [49]. The granulation tissue is composed of blood vessels which are situated in connective tissue composed of mesenchymal cells and leukocytes [49]. Following extraction, blood interacts with the exposed endothelial cells and extracellular matrix, which leads to the accumulation of platelets and subsequent fibrin clot formation [51]. The blood clot also acts as an adhesive structure for cells that will play a further role in the next stages of alveolar healing [51]. Together, the blood clot and activated platelets, along with endothelial cells and leukocytes, release cytokines and growth factors that control the next stage of alveolar healing—the inflammatory stage [51]. The inflammatory stage begins within 48–72 h following extraction. The released cytokines and growth factors trigger the recruitment, migration, differentiation, and proliferation of inflammatory cells [48]. Neutrophils are numerous at this point, along with leukocytes, and monocytes which are converted to macrophages [52]. During this stage, specific growth factors activate fibroblasts and osteoblasts, necessary for the progression of alveolar healing [53]. Furthermore, the fibrin clot is replaced with granulation tissue, which contains many new blood vessels, along with inflammatory cells and immature fibroblasts, which form the connective tissue [48,49]. Thirdly, the proliferative stage begins around 2 weeks post-extraction. It is marked by fibroplasia, which constitutes the rapid development of the provisional matrix, which replaces the granulation tissue and any remnants of periodontal ligaments [51]. The provisional matrix is made up of mesenchymal cells localized in a connective tissue matrix [49]. This stage has two parts: fibroplasia and woven bone formation proceeded by setting the woven bone around the blood vessels [48,51]. The woven bone is formed by osteoprogenitor cells in the abundance of blood vessels within the provisional matrix [48,51]. The literature suggests that granulation tissue may be replaced by woven bone within 6 to 8 weeks post-extraction [49]. The last stage, modeling and remodeling, occurs from 4 weeks following dental extraction. At this time, the woven bone is replaced by mature bone [48,51]. From the fourth week following extraction, mature mineralized trabeculae bone and marrow form in the apical aspect of the alveolus, and in the coronal area around 12 weeks later, resulting in total sealing of the alveolus with cortical bone [54]. The histological stages of granulation tissue formation and healing of the alveolus have not yet been specifically described in horses. We can observe that there is no clear definition of when an alveolus is considered “fully healed”, and each discussed study has created their own interpretation of this term. Healing times will differ based on certain factors, such as the age of the horse, the extraction method, and complications during the extraction procedure or post-extraction. Based on the most detailed descriptions available in the literature [15,16,23], complete alveolar healing may be considered achieved when the alveolus is completely sealed with granulation tissue [15] and the granulation tissue is lined with normal gingival tissue [16,23]. At this stage, placement of an alveolar plug may no longer be beneficial.

Limone et al. [12] used PVS impression material to plug the alveolus and formed it specifically to allow space for blood clot formation in the apical one-third of the alveolus. During this study, recheck examinations occurred 7, 21, and 42 days following extraction. At this time, the PVS plugs were digitally removed, the alveolus was inspected, and the same plug was inserted back into the alveolus. However, before replacement, the apical aspect of the plug was trimmed to allow for further granulation tissue formation at the apical aspect of the alveolus. After 42 days, the PVS plugs were removed and not replaced, though the extent of alveolar healing was not described at this point.

Dixon et al. [10] successfully utilized alveolar plugs in situations where an active discharging tract was present after tooth extraction resulting from mandibular or rostral maxillary cheek teeth apical infections. In these cases, the draining tract and the apical part of the alveolus were irritated and flushed. Next, an alveolar plug, in the form of a dental wax or PMMA mass, was placed 2–4 cm into the alveolus [10]. This allowed the draining tract to remain clean and granulate closed. If the alveolus is shallower, these plugs are displaced more easily and a sturdier material, such as PoP, may be considered [8,11].

There are also circumstances when one type of alveolar plug material is used at first; however, if it is not efficient at protecting the alveolus from contamination, then a different material may be elected. For example, Gergeleit and Bienert-Zeit [21] changed intra-alveolar gauze swabs (infused with medical grade honey or metronidazole) to PVS when they noted that the gauze swabs did not adequately prevent food contaminating the alveolus. Similarly, changing the alveolar plug material during the healing process may be beneficial once a sufficient amount of granulation tissue fills the alveolus.

### 5.2. Post-Extraction Complications

Among the summarized case reports and research articles, each study reported more or less clinically significant post-extraction complications, which may be caused by the original dental disease, extraction procedure, or plug application. Beginning with plug-related complications, the following problems were reported: problem with occlusion [15], plug embedded in granulation tissue resulting in difficulties of plug removal [8,9,10], plug loosening [10,13,20,22], plug displacement to the oral cavity [11,16,20,23] or sinus [13,17], and alveol contamination with food material [8,13,21]. On the other hand, delayed alveolar granulation [8,9,20] and fistula formation [12,16] may be related to pre-existing apical infection [8,9,10,11,12,13,14,15], periodontal infection [8,11,15], suturitis [15], fractures of the tooth [8,10,18], fractures of the mandible [16], or the presence of retained tooth root fragments within the alveolus [7,16,22] or sinus [18]. Sinusitis may be considered as a complication due to both—being a reason of tooth extraction resulting from apical infection [14,15,16,17,18] or being a consequence of tooth extraction [8,10,27]. Also, sequestration may be related to pre-existing bone sequestrae, resulting in tooth extraction [16], or developed post-extraction as a consequence of trauma following complicated extraction [7,9,10,15,21]. Similarly, abscess formation [21], draining tract formation to the bone [16], and some fistulae—including both oro–nasal [10,20], oro–sinus [7,8,9,10,13,14,17,18,27], and oro–cutaneous [8,15,18,21] fistulae—may be a consequence of trauma following complicated extraction. The most commonly seen post-extraction complications of equine cheek teeth occur after challenging procedures or following disturbances to the post-extraction blood clot [9]. In horses, it is possible that some cases exhibit post-extraction blood clot infection, which aggravates the alveolus and leads to alveolar infection [9]. Moreover, in this species, it is highly unlikely that the proper healing of the alveolus is solely dependent on the presence of a blood clot that fills up the extensive alveolus. During mastication, large pressure is exerted on the teeth, and the force of chewing highly fibrous material would displace the blood clot from the alveolus [9].

Turek et al. [20] were presented with two common problems involving the alveolar plug during the treatment of the persistent oro–nasal fistula in a two-year-old horse. Firstly, the PVS plug was likely inserted too high into the alveolus, which prevented granulation tissue formation, thus impacting the closure of the fistula. Later, once the diameter of the fistula was smaller, it was difficult to maintain the PVS plug in the alveolus, leading to contamination of the nasal cavity with feed. Upon modification and attachment of the plug to the maxillary bone using nylon thread, the plug was maintained in the alveolus for a longer amount of time. PVS was found to be the only material that could be fixed in this way, while still allowing it to execute its function. Contrastingly, instead of the alveolar plug being displaced from the alveolus too soon, Dixon et al. [10] described one patient where the alveolar plug became incorporated into the granulation tissue in the alveolus. In other cases, observed by Dixon et al. [10], quidding became apparent in two horses a few months after oral extraction. Following oral cavity examination, this was found to be caused by sharp edges of the loose PMMA alveolar plugs, which caused buccal pain. Once these plugs were removed, symptoms ceased. Delayed alveolar granulation following tooth extraction was observed by Caramello et al. [8] in 13% of cases. Interestingly, this complication was not found to be associated with the type of alveolar packing used but rather was due to the presence of an alveolar bone sequestrum, persistent sepsis, the presence of a fistula, and persistent tooth fragments that remained in the alveolus [8]. Additionally, the partial embedding of gauze swabs into the alveolar granulation tissue was another limitation of alveolar plugs observed in three horses during re-examinations [9]. Despite these residual gauze fibers that were removed, the alveoli continued to completely heal, and further clinical issues were not observed [9].

Sometimes, depending on the alveolar packing material chosen and the way it settles in the alveolus, it may be difficult to remove. Caramello et al. [8] encountered 2 out of 15 horses where PMMA was chosen as the alveolar packing material, and in one of these horses, to specifically close the space between the sinuses and the oral cavity. As a result of the horses’ nature and the type of packing material used in these two cases, the horses had to be put under general anesthesia for plug removal. Therefore, it is critical to select the appropriate packing material for each individual alveolus, together with correct application, so it can be removed without damaging surrounding tissues, while ensuring patient safety.

#### 5.2.1. Managing Oro–Sinus/Nasal Fistulae

Turek et al. [20] used PVS to plug a long-lasting fistula between the nasal and oral cavity following extraction of deciduous teeth 607 and 608, Triadan 208, and an ossifying fibroma from the left nasal cavity. To prevent displacement of the alveolar plug in this case, the authors modified the standard PVS plug by adding a plastic sleeve into the PVS packing material and attaching a nylon plastic thread, which made it possible to fasten the plug to the maxillary bone surface. This technique, along with the application of an autologous bone graft, provided complete closure of the oro–nasal fistula. In some cases, improper handling of the PVS mass and excessive use of force while pushing the mass into the dental alveolus resulted in pushing a fragment of the mass directly into the paranasal sinuses [17,34]. Likewise, if the horse experienced dental sinusitis, the apical aspect of the alveolus was examined by digital exploration, and a gauze swab or PMMA mass was placed 2–3 cm into the alveolus to seal it [7].

Oro–sinus/nasal fistulae were also encountered as post-extraction complications by Kennedy et al. [9]. These cases were successfully treated by PMMA plugs placed into the dental alveolus. PMMA plugs provide a more robust barrier from the oral cavity, giving the fistulae time to heal. Hawkes et al. [55] similarly demonstrated the effectiveness of using permanent PMMA prosthesis to treat persistent oro–maxillary fistulae in horses. A persistent oro–sinus fistula, whose diameter did not change at the 2.5 month and 8 month re-examinations, was protected using PMMA and dental impression compound plugs. This provided protection from the oral cavity while allowing transnasal endoscopic–guided debridement of the sinus part of the fistula using endoscopic forceps. Granulation tissue formation and progression could be observed through the endoscope without needing to remove the alveolar plug material. The main disadvantage of this material may be its hardness and lack of elasticity, which may limit and block the formation of granulation tissue and healing of the alveolus.

#### 5.2.2. Secondary Dental Sinusitis

Both Stemmet et al. [17] and Turek et al. [34] described horses which developed severe secondary sinusitis following tooth extraction, due to displaced PVS plugs from the dental alveolus into the sinus. Specifically, Stemmet et al. [17] reported a young Thoroughbred mare that experienced severe, chronic, unilateral sinusitis of the left rostral maxillary sinus after Triadan 210 extraction two months prior. Further inspection of the head using radiographs revealed a relatively large, well circumscribed soft tissue opacity localized in the left rostral sinus compartment [17]. Computed tomography examination displayed accumulation of fluid and gas attenuation material in the left frontal, rostral and caudal maxillary, and ventral conchal sinuses [17]. The foreign structure was visualized in the caudal area of the rostral maxillary sinus. A left frontal sinus trephination portal was created through which a 1.4 m video endoscope was able to visualize a blue mass with rubber consistency in the rostral maxillary sinus, consistent with the foreign body found in the computed tomography examination, agreeing to the presence of the alveolar plug in the rostral maxillary sinus [17]. Given the complications arising from displacement of the alveolar plug and the absence of regular assessment of its position, it can be concluded that the alveolar plug needs to be monitored regularly. Significant attention needs to be given to alveolar plugs placed in alveoli following extraction of maxillary teeth 08 to 11, as the displacement or loss of the respective alveolar plug will most likely lead to oral sinus fistula formation if left unchecked [17].

## 6. Recommended Management of the Equine Alveolus Post Extraction

Ultimately, at this time, there is no objective data on the optimal management protocol of equine post-extraction alveoli, including the best type of plug material, the depth of insertion of the plug material into the alveolus, or the frequency of alveolar examination and plug replacement. Dixon et al. [10] initially did not recommend routine post-extraction examination of the oral cavity and alveolus following cheek tooth extraction, citing Tremaine [56], who reported no complications in 49 horses after cheek tooth extractions. Conversely, horses in their study experienced post-extraction complications, including a case with a gauze swab embedded in alveolar granulation tissue and alveolar sequestration, which was a common issue following cheek tooth extraction [10]. However, gauze swabs may be an effective alveolar packing material, providing alveolar drainage and less resistance for sequestrae separation [21]. Similarly, Gergeleit and Bienert-Zeit [21] also observed that packing the alveolus with gauze swabs may be more efficient in alveolar healing when a fistula is not present, compared to PVS plugs. However, post-extraction complications were also observed with the use of gauze swab plugs, which hindered the healing of the alveolus [9,10,13,21], including cause alveolar contamination with food materials [21].

In particular, mandibular alveoli exhibit a higher probability of developing post-extraction problems, therefore it is recommended to control the healing process of these alveoli following extraction, including removal of sequestrae and repeated, even on a weekly basis, examinations and replacement of alveolar plugs until the alveolus is fully healed [9]. Likewise, proper application of an alveolar plug to protect an oro–maxillary/nasal fistula is critical, as the loss of the plug can risk the renewal of sinus contamination with food particles from the oral cavity and consequently, sinusitis [18]. Overall, anecdotal evidence shows that simply packing the alveolus post-extraction and leaving the material in the alveolus until it is spontaneously displaced is insufficient for proper healing [24]. Although this is important following the extraction of every cheek tooth, it is especially important following the extraction of Triadan 06–08 mandibular cheek teeth, notably in younger horses, when the alveoli can be >9 cm deep [9,21]. Once the tooth is removed, the alveolar plug should be placed in the alveolus immediately and then should be replaced following 7–10 days [24]. At this time, the alveolus should be examined visually with a dentalscope or mirror and digitally, to evaluate granulation tissue formation over the surfaces of the alveolus [24]. Otherwise, rough parts found in the alveolus may signify alveolar sequestration or “dry socket” [24]. If mobile, the sequestrae can be removed at this time [24]. Some sequestrae may take 3–4 weeks to loosen from the alveolus. Otherwise, upon examination, if the alveolus is healing properly, it should be lightly flushed, keeping the blood clot in place [24]. A new alveolar plug, smaller in size than the one removed, should be placed into the top one-third of the healing alveolus. After around 7–14 days, the alveolus should be reassessed for proper granulation tissue formation and for the presence of delayed sequestration. Follow-up examinations may be considered at this time interval until the alveolus is significantly filled with granulation tissue and can be left without an alveolar plug. The healing end-point considered in each study also differed between discussed studies. Based on this information gathered from the literature, defining an end-point for alveolar healing may also be beneficial when deciding whether an alveolus needs to be repacked or is at a safe stage to be left without an alveolar plug.

It is important to note that following extractions due to apical infection, particularly in younger horses, the alveolus should be checked and tended to more rigorously for a longer period of time [24]. The same should be practiced for an alveolus that contains oro–nasal [10,16,20], oro–sinus [7,8,9,10,13,14,16,17,18,27], or oro–cutaneous [8,12,15,16,18,21] fistulae, whether from disease or following repulsion during extraction. In these cases, if the alveolar plug is dislodged from the alveolus for a longer period of time, the tracts may epithelialize and turn into fistulae [24]. Post-extraction alveolar flushing with water [15,16,21], normal saline [12,20], or Hartmann’s solution [7] has been recommended to remove feed material from the alveolus. Furthermore, flushing of healing alveoli has been described following both routine oral extraction [14] and complicated extractions performed by repulsion techniques, particularly ostectomy [12] or trephination [20]. After routine extraction, alveolar flushing was performed at 6-day intervals [14], whereas following complicated extraction, it was performed at intervals of 1–7 days (after 3, 4, 9, and 16 days, and then once weekly) [20] or 7–21 days (after 7, 21, and 42 days) [12]. In studies describing both routine and complicated cases, alveolar flushing during post-extraction rechecks was performed every 7–10 days [16], after 10–14 days [9], or every 2–4 weeks [10]. Therefore, it may be cautiously suggested that, when feasible, inspection of the alveolus [7,9,10,12,15,16,17,21,27], including gentle flushing [9,10,12,16], should be performed every 7–14 days post-extraction until the alveolus is completely filled with granulation tissue [15] and the granulation tissue becomes lined with normal gingival tissue [16,23]. After complicated extractions [7,8,9,10,11,12,13,16,17,18,20,21,22,23,27] or cases with complications [7,8,9,10,11,12,13,14,15,16,17,18,20,21,22,23,27], the frequency of alveolar inspection [11,20,21], alveolar flushing [20], and plug replacement [20,21] may need to occur more frequently. However, the variation in the frequency of post-extraction examinations described in the summarized studies makes it difficult to formulate clear recommendations. Therefore, further research is needed to establish guidelines to optimize post-extraction alveolar management in horses, in turn, reducing post-operative complications following equine cheek tooth extraction.

## 7. Limitations

The main limitation of this review arises from the lack of a systematic evaluation of the available literature, which ideally should have been conducted in accordance with the Preferred Reporting Items for Systematic Reviews and Meta-Analyses (PRISMA) guidelines [57]. Consequently, this narrative review lacks a formal risk-of-bias assessment and quantitative data synthesis, both of which could have strengthened the evidence-based evaluation of the alveolar plugs used. It may be observed that the majority of the retrieved records consisted of case series [7,8,10,11,13,16,21,27] and case reports [12,14,17,18,20,22], whereas only two studies had a cohort design [9,23].

Furthermore, one cohort study was retrospective [9] and the other prospective [23], and both investigated substantially different aspects of the types and use of alveolar plugs following equine cheek tooth extraction, making direct comparison unjustified. Therefore, the evidence supporting the recommendations discussed in this review is primarily clinical rather than strongly evidence-based. This is particularly evident in recommendations suggesting that the coronal one-third of the alveolus should be filled with packing material [10,16,17,34], or that complete sealing of the alveolus with granulation tissue [15] and subsequent lining of the granulation tissue with normal gingival tissue [16,23] should be considered indicators of complete healing. Consequently, the optimal depth of plug placement and the criteria defining complete alveolar healing require further investigation in studies with stronger scientific evidence.

Another limitation of this review is the high heterogeneity of the summarized studies. Not only were there no directly comparable studies, but there was also a lack of standardized protocols describing post-extraction alveolar healing. This limitation became particularly evident when comparing healing times and recheck intervals, making it difficult to formulate definitive clinical recommendations.

## 8. Conclusions

Given that most of the reviewed studies consisted of descriptions of clinical cases, it may be concluded that the use of alveolar plugs may be considered clinically useful following cheek tooth extraction in horses, particularly in selected cases at risk of contamination, delayed granulation, or fistula formation. Selecting an appropriate alveolar packing material for each individual case may be crucial for minimizing complications during the healing process, and a well-adjusted plug may play an important role in protecting the deeper alveolar tissues from unnecessary pressure and contamination. Although standardized protocols for equine alveolar plug management have not yet been established, it may be suggested that any of the currently described packing materials can be used following routine tooth extractions. For marginally positioned teeth, gauze swab plugs may be more favorable; however, these plugs may not be the optimal choice in older horses with shallow alveoli. Regardless of whether the extraction is routine or complicated, alveolar inspection at 7–14-day intervals may be recommended. Nevertheless, prospective comparative studies are needed to determine the optimal plug material, insertion depth, replacement interval, and follow-up protocol for different extraction sites and clinical scenarios. Moreover, defining the end-point of alveolar healing requires further histological investigation, as it is currently considered clinically achieved when the alveolus is completely sealed with granulation tissue and the granulation tissue is lined with normal gingival tissue. At this stage, placement of an alveolar plug may no longer be beneficial.

## Figures and Tables

**Table 1:** Summary of case reports on the alveolar healing process involving the use of plug materials in equine cheek teeth.

Study	Study Type	Packing Material	Eluted Medications	Tooth Location *	Extraction Method	Alveolar Management	Recheck Intervals	Healing Time	Alveolar Healing End-Point	Complications
Horbal et al., 2018 [22]	Case report/ complicated (1 horse)	Gauze swabs	Normal saline	1 mandibular cheek tooth-root fragments (410)	Curettage (1 tooth root)	Not reported	After 3 weeks	11 months	“Normal healing” with no specification	Plug loosening
Nottrott et al., 2018 [18]	Case report/ complicated (1 horse)	PVS	None	1 maxillary cheek tooth (208)	Repulsion (sinusotomy, 1 tooth)	Rechecks: debridement (if fistula present)	After 10 weeks, then 2 months later, 3 months later, and 14 months later	Not reported	Alveolus: not reported; fistula: complete closure	Oro–sinus fistula, sinusitis, oro–cutaneous fistula
Limone et al., 2020 [12]	Case report/ complicated (1 horse)	PVS	None	1 mandibular cheek tooth (309)	Repulsion (ostectomy, 1 tooth)	Post-extraction: flushing, debridement	After 7, 21, and 42 days	3 months	“Normal healing” with no specification	Oro–cutaneous fistula
Kau et al., 2021 [14]	Case report/routine (1 horse)	Gauze swabs	Iodoform	1 maxillary cheek tooth (209)	Oral extraction (1 tooth)	Post-extraction: debridement/rechecks: flushing (unless blood clot present), debridement (if fistula present)	After 4 days, then 6-day intervals until discharge at day 16	Phone call 1 week after discharge, then at 18 months	Alveolus: good granulation with no specification; fistula: complete closure	Oro–sinus fistula, sinusitis
Turek et al., 2021 [20]	Case report/ complicated (1 horse)	PoP, PVS	Iodoform	1 maxillary cheek tooth (208)	Repulsion (trephination, 1 tooth)	Post-extraction: flushing, debridement/rechecks: flushing, debridement (including fistula)	After 3, 4, 9, and 16 days then once per week	7 months	Alveolus: not reported; fistula: complete closure	Oro–nasal fistula, plug loosening, plug displacement to oral cavity, delayed alveolar granulation
Stemmet et al., 2022 [17]	Case report/ complicated (1 horse)	PVS, gauze swabs and PVS	None	1 maxillary cheek tooth (210)	Repulsion (trephination, 1 tooth)	Rechecks: debridement	After 3 weeks, then 2 weeks later, then 2 months later	Not reported	Not reported	Oro–sinus fistula, sinusitis, PVS plug displacement to sinus
McQuillan et al., 2024 [15]	Case report/routine (1 horse)	PMMA, gauze swabs, PVS	Gauze swabs with povidone–iodine	2 maxillary cheek teeth (107, 109)	Oral extraction (2 teeth)	Post-extraction: flushing	Weekly	6 weeks	Alveolus: complete seal of granulation	Oro–cutaneous fistula, suturitis, sequestration, occlusal leakage when PMMA used (PMMA replaced with PVS)

PMMA—polymethyl methacrylate, PoP—plaster of Paris, PVS—polyvinyl siloxane, * the Triadan system used.

**Table 2:** Summary of research articles on the alveolar healing process involving the use of plug materials in equine cheek teeth.

Study	Study Type	Packing Material	Eluted Medications	Tooth Location *	Extraction Method	Alveolar Management	Recheck Intervals	Healing Time	Alveolar Healing End-Point	Complications
Prichard et al., 1992 [13]	Research article (case series; level 4 EBMR) /complicated (61 horses)	Dental wax, gauze swabs, PVS	None	77 cheek teeth: 50 maxillary teeth (06–09); 27 mandibular teeth (06–10)	Repulsion (77 teeth) (19 flap sinusotomies, 11 trephinations, 5 preexisting fistula, 1 unrecorded approach)	Not reported	Not reported	Not reported; plugs left for spontaneous displacement by the granulation tissue	Not reported	Oro–sinus fistula, sinusitis, plug displacement to sinus, alveolus contamination with food, plug loosening
Trostle et al., 2000 [11]	Research article (case series; level 4 EBMR) /routine and complicated (8 horses)	PoP	Metronidazole, cefazolin, ampicillin with sulbactum or trimethoprim sulfa	8 maxillary cheek teeth (06–09)	Oral extraction (2 teeth)/repulsion (sinusotomy or trephination, 6 teeth)	Not reported	After 1 day	Not reported	Not reported	Plug displacement into oral cavity
Dixon et al., 2005 [10]	Research article (case series; level 4 EBMR) /routine and complicated (100 horses)	PMMA, gauze swabs, dental wax	Gauze swabs with povidone iodine or metronidazole	111 cheek teeth: 60 maxillary teeth (06–11); 51 mandibular teeth (06–11, supernumerary)	Oral extraction (102 teeth)/repulsion (unrecorded approach, 9 teeth)	Post-extraction: curettage, digital palpation, flushing/rechecks: flushing (if fistula present)	None for the initial 39 cases, later cases recommended recheck after 2–4 weeks	Not reported; few weeks to several months depending on complications	Not reported	Oro–nasal fistula, oro–sinus fistula, sinusitis, sequestration, gauze swab embedded in granulation tissue, plug loosening
Vlaminck et al., 2006 [23]	Research article (prospective cohort study; level 2 EBMR) /complicated (5 horses)	PVS	PVS with bone substitute (or PVS alone)	10 maxillary cheek teeth (all 08s)	Repulsion (flap sinusotomy, 10 teeth)	Not reported	Monthly for 1 year, then at the end of year 2	2 months	Granulation lined by normal gingival tissue	Plug displacement into oral cavity
Earley et al., 2013 [16]	Research article (case series; level 4 EBMR)/ complicated (5 horses)	(a) PVS (b) PVS (c) Gauze swabs, PoP, PMMA, PVS (d) PVS (e) PVS	(a) None (b) None (c) Gauze swabs with scarlet oil or povidone–iodine (d) None (e) None	7 cheek teeth: 4 maxillary teeth; 3 mandibular teeth, including: (a) 108, 109 (b) 209 (c) 108 (d) 408 (e) 406, 407	(a) Repulsion (osteotomy, 2 teeth) (b) Repulsion (trephination, 1 tooth) (c) Not reported (d) Repulsion (osteotomy, 1 tooth) (e) Repulsion (buccotomy, 2 teeth)	(a) Post-extraction: curettage (b) Not reported (c) Post-extraction: flushing/rechecks: flushing (always), debridement (if fistula present) (d) Post-extraction: curettage (e) Not reported	(a) Not reported (b) 1,2,11 months (c) every 7–10 days (d) 6 months (e) Not reported	(a) Not reported (b) 11 months (c) Not reported (d) Not reported (e) 6 months	(a) Not reported (b) Granulation lined by normal gingival tissue (c) Not reported (d) Not reported (e) “Normal healing” with no specification	(a) Oro–sinus fistula, sinusitis (b) Oro–sinus fistula, sinusitis, plug displacement into oral cavity (c) Oro–nasal fistula, plug displacement to oral cavity (d) Draining tract to bone (e) Oro–cutaneous fistula
Caramello et al., 2020 [8]	Research article (case series; level 4 EBMR)/ routine and complicated (137 horses)	None, PMMA, PoP, gauze swabs, PVS	Gauze swabs with zinc oxide– iodophor– petrolatum	162 cheek teeth: 117 maxillary teeth (06–11), 45 mandibular teeth (06–11)	Oral extraction (68 teeth)/repulsion (trephination, 52 teeth; flap sinusotomy, 26 teeth; buccotomy, 16 teeth)	Not reported	Not reported	Less than 2 weeks to over 2 months	Not reported	Oro–sinus fistula, sinusitis, oro–cutaneous fistula, delayed alveolar granulation, alveolus contamination with food, difficulties to remove PMMA plug
Gergeleit and Bienert-Zeit, 2020 [21]	Research article (case series; level 4 EBMR)/ routine and complicated (20 horses)	Gauze swabs, PVS	Gauze swabs with medical grade honey or metronidazole	20 mandibular cheek teeth (06–11)	Oral extraction (13 teeth)/ tooth sectioning (2 teeth)/ repulsion (preexisting fistula, 3 teeth; buccotomy, 2 teeth)	Post-extraction: flushing/rechecks: digital palpation, oroscopical view	After 2 days, then weekly until almost complete alveolar healing	2–5 months	Almost complete “normal healing” with no specification	Oro–cutaneous fistula, abscess, sequestration, alveolus contamination with food (when gauze swabs were used)
Kennedy et al., 2020 [9]	Research article (retrospective cohort study; level 3 EBMR)/ routine and complicated (400 horses)	PMMA, gauze swabs	Gauze swabs with metronidazole, cloxacillin benzathine, or ampicillin	428 cheek teeth: 290 maxillary teeth (06–11, supernumerary), 138 mandibular teeth (06–11)	Oral extraction (343 teeth)/repulsion (trephination, 46 teeth; transbuccal screw extraction, 39 teeth)	Rechecks: digital palpation, flushing	After 10–14 days	Not reported; few weeks to several months depending on complications	Not reported	Oro–sinus fistula, sinusitis, gauze swab embedded in granulation tissue, sequestration, non-healing post-extraction alveoli
Kamps and Barakzai, 2024 [7]	Research article (case series; level 4 EBMR)/ complicated (19 horses)	PMMA	None	20 cheek teeth: 15 maxillary teeth (07–10), 5 mandibular teeth (07–09)	Repulsion (trephination, 13 teeth; flap sinusotomy, 7 teeth)	Post-extraction: flushing, curettage	After 14 days, if plug still in place; per 4–6 weeks	Mean 29 months (6–48 months)	Not reported	Oro–sinus fistula, sinusitis, sequestration, intra-alveolar fragment causing masticatory difficulties
Leps et al., 2024 [27]	Research article (case series; level 4 EBMR)/ complicated (29 horses)	PMMA, PVS	None	29 cheek teeth: 27 maxillary teeth (109–111, 207, 209, 210), 2 mandibular teeth (309)	Tooth sectioning (29 teeth)	Post-extraction: fistula only curettage	Every 2 weeks	2–9 weeks	Not reported	Oro–sinus fistula, sinusitis

EBMR—Evidence-Based Medicine Rating, PMMA—polymethyl methacrylate, PoP—plaster of Paris, PVS—polyvinyl siloxane, * the Triadan system used.

**Table 3:** Characteristics of packing materials used as alveolar plugs in equine dentistry.

Packing Material	Elute Medications	Biodegradability	Remaining Advantages	Remaining Disadvantages
PMMA	Yes	No	pliable, quick-setting, durable, cost-effective	exothermic curing, hard
PoP	Yes	Yes	cold curing, cost-effective	longer period of time to harden/set
Dental wax	No	No	malleable, chemically neutral material, cost-effective	may not remain in the alveolus for an extended period of time
Gauze swabs	Yes	No	absorbs and drains purulent discharge, soft, cost-effective	frequent changes, less effective at preventing feed impaction
PVS	No	No	flexible, high tensile strength, easily contoured to fit the alveolus	expensive

PMMA—polymethyl methacrylate, PoP—plaster of Paris, PVS—polyvinyl siloxane.

## Data Availability

The original contributions presented in this study are included in the article. Further inquiries can be directed to the corresponding authors.
